# Stay social, stay young: a bioanthropological outlook on the processes linking sociality and ageing

**DOI:** 10.1007/s11357-024-01416-5

**Published:** 2024-11-11

**Authors:** Vincenzo Iannuzzi, Nicolas Narboux-Nême, Andrea Lehoczki, Giovanni Levi, Cristina Giuliani

**Affiliations:** 1 Laboratory of Molecular Anthropology & Centre for Genome Biology, Department of Biological, Geological and Environmental Sciences, University of Bologna, Via Selmi 3, 40126 Bologna, Italy; 2 Physiologie Moléculaire Et Adaptation, CNRS UMR7221, Département AVIV, Muséum National d’Histoire Naturelle, Paris, France; 3 Doctoral College, Health Sciences Program, Semmelweis University, Budapest, Hungary; 4 Institute of Preventive Medicine and Public Health, Semmelweis University, Budapest, Hungary

**Keywords:** Sociality, Ageing, Lifespan, Human evolution, Physiology, Stress

## Abstract

In modern human societies, social interactions and pro-social behaviours are associated with better individual and collective health, reduced mortality, and increased longevity. Conversely, social isolation is a predictor of shorter lifespan. The biological processes through which sociality affects the ageing process, as well as healthspan and lifespan, are still poorly understood. Unveiling the physiological, neurological, genomic, epigenomic, and evolutionary mechanisms underlying the association between sociality and longevity may open new perspectives to understand how lifespan is determined in a broader socio/evolutionary outlook. Here we summarize evidence showing how social dynamics can shape the evolution of life history traits through physiological and genetic processes directly or indirectly related to ageing and lifespan. We start by reviewing theories of ageing that incorporate social interactions into their model. Then, we address the link between sociality and lifespan from two separate points of view: (i) considering evidences from comparative evolutionary biology and bioanthropology that demonstrates how sociality contributes to natural variation in lifespan over the course of human evolution and among different human groups in both pre-industrial and post-industrial society, and (ii) discussing the main physiological, neurological, genetic, and epigenetic molecular processes at the interface between sociality and ageing. We highlight that the exposure to chronic social stressors deregulates neurophysiological and immunological pathways and promotes accelerated ageing and thereby reducing lifespan. In conclusion, we describe how sociality and social dynamics are intimately embedded in human biology, influencing healthy ageing and lifespan, and we highlight the need to foster interdisciplinary approaches including social sciences, biological anthropology, human ecology, physiology, and genetics.

## Introduction

The proportion of elderly individuals is rapidly increasing in the world and is expected to reach about 16% of the population in 2050 [1], exacerbating the burden on the medical system due to the higher prevalence of diseases linked to ageing such as dementia, cardiovascular problems, osteoporosis, and sarcopenia [2]. Sociality is one of the main factors associated with better health and longevity [3]; social isolation has an impact on mortality equivalent to recognized risk factors such as obesity, smoking, lack of physical activity, and poor access to health care [4, 5]. It has been shown that social isolation and loneliness are associated with an increase in the likelihood of mortality of 29% and 26% respectively [6]. It appears that social organization, social interactions, and intergenerational transfer are associated with delayed ageing, reduced mortality, and increased longevity not only in humans but also in other mammals [7–11]. These observations suggest the existence of evolutionary-conserved biological processes associating sociality with longevity. Comprehension of these processes could have profound effects on the understanding of human social dynamics with a direct impact for decision-makers to optimize the support people at this stage of life, for them and society [1].

**Fig. 1:**
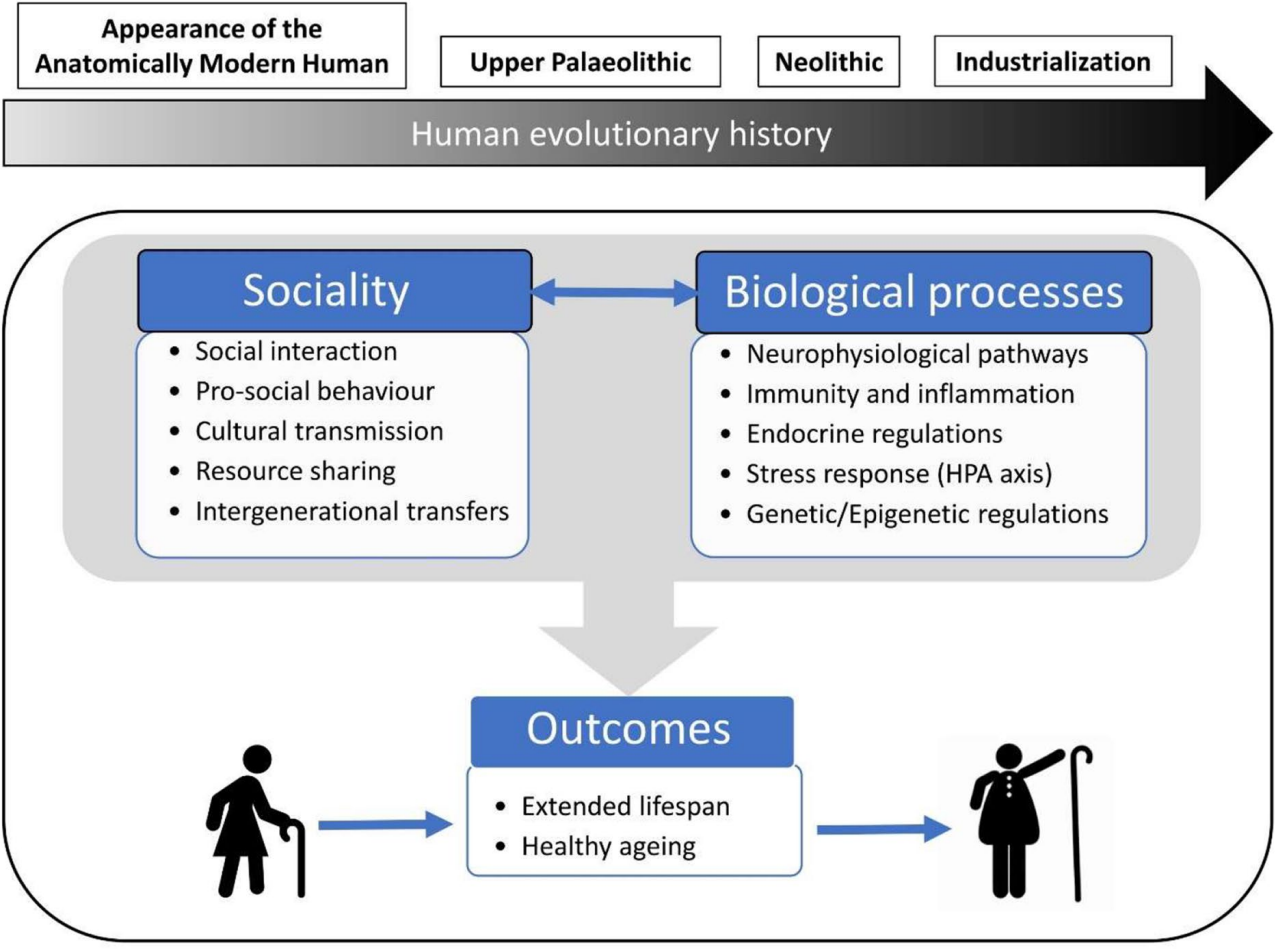
Schematic representation of the narrative approach adopted in this review. The approach is based on the analysis of the interplay between sociality and biological processes that are central to the process of healthy ageing and that help to explain the extended lifespan that is a characteristic of our species. The relationship between these factors has been subject to continuous modulation throughout the course of human evolution

This review summarizes recent advances in biological anthropology, physiology, epigenetics, and genetics to highlight how the relationship between sociality and biological processes has shaped human lifespan throughout evolutionary history and continues to impact it in contemporary populations (Fig. 1). First, we describe theories of human ageing including social aspects, then we provide insight into bioanthropological evidence that establishes a connection between sociality and natural variations in lifespan across different timescales. Among various factors, we explore the impact of inter-generational transfer and resource sharing on life history traits of pre-industrial societies and indigenous populations, where the influence of the “modern” environment is reduced. Indeed, extended lifespans are not exclusive to contemporary human life: humans exhibit the longest lifespan among primates, even in conditions characterized by high mortality, such as those experienced by hunter-gatherers. Then, we present an analysis of how social dynamics influences physiological, neurological, and endocrinological processes associated with lifespan determination, providing examples from genetically modified model animals. We show that exposure to chronic social stressors can permanently deregulate neurophysiological and immunological pathways, promoting accelerated ageing. In conclusion, we describe genetic and epigenetic factors involved in the link between sociality and lifespan and we suggest the use of biomarkers of ageing such as the “epigenetic clocks” in study aimed at investigating the link between sociality and human ageing. We conclude that multidisciplinary approaches including social sciences, biological anthropology, ecology, physiology, and genetics are needed to decipher the multiple facets of an ageing society and to be prepared to face the ongoing demographic shift.

## Theories of human ageing which include social aspects

Over 300 theories have been proposed regarding the ageing process [12]—see for example: [13–16]. However, only a few of them integrate the impact of social dynamics or pro-social behaviours or social isolation while these aspects are progressively becoming the central focus in contemporary outlooks to “Geroscience Research” [17].

We begin by discussing major classical theories of ageing which include fundamental social concepts such as intergenerational transfer and resource sharing.

### Kin selection theory

One of the first attempts to include the social dimension in the study of lifespan extension is the so-called kin-selection theory [18–21]. According to this theory, the lifespan of an individual may negatively or positively affect the fitness of its relatives, creating an additional selective pressure on longevity [22]. In this light, a delayed onset of ageing might increase the quality and the quantity of care provided to the offspring and to the closest relatives, showing that life-history evolution is influenced by inclusive fitness effects. Although the “kin-selection theory” has been questioned for some of its applications [23, 24], it is widely accepted as an explanation for social behaviours observed in many taxa [25–28]. Indeed, the “kin-selection theory” has been widely investigated in eusocial species, in mammals, and in humans.

In the case of eusocial insects, the prediction that reproductive altruism should be directed toward kin is firmly supported [29]. In ants, for instance, the long living queens caste is an example of a phenotype evolved thanks to kin selection [18, 30, 31]. In fact, the caste system of division of labour between short living sterile workers and long living queens (which share the same genome) allows only the latter to reproduce and thus to pass their genes to the next generation. Since the queen is the sole reproductive individual in these monogynous societies, sterile workers can avoid the demise of the entire colony only by helping their fully fertile relative to survive and reproduce. This leads to strong selection for queen longevity [32, 33].

Kin selection is a driver of longevity also in mammalian species. In a recent study, Zhu and co-workers [34] have performed a comparative phylogenetic analysis of the longevity of about 1000 mammalian species living in three different modes of social organization: solitary, pair-living, and group-living. Their main finding is that group-living species live longer than solitary species; this result is valid both in terms of “absolute longevity” and of “residual longevity” (after adjustment for body mass). Group-living species may live longer due to reduced risks of predation and/or starvation. Furthermore, in primates, it has been shown that stable social bonds in a group are associated to prolonged lifespan [35–37]. Thus, association among kins can influence coalition in a group, cooperative breeding, and the emergence of social hierarchies which, ultimately, can increase the evolutionary fitness of a social species. This study provides also results from brain transcriptomic analyses of 94 mammalian species showing that hormonal regulation and immunity constitute a shared mechanism that link social organization and longevity [34]. In view of the biological mechanisms summarized later in this review, this observation is not surprising, it is, however, in the brain that one has to finally find the common genetic regulations that affect social behaviour and longevity.

In humans, the general framework of the kin-selection theory includes also the “grandmother hypothesis” [38], and the “intergenerational transfers” model [39] that Bourke (2007) attempted to reconcile.

#### Grandmother hypothesis

The “grandmother hypothesis” [38] dates back to the 1960s and was then revised by Hawkes and colleagues [40]. This theory states that extended post-menopausal lifespan coevolved with the mother–child food-sharing habit, a practice that helps elder females to improve their daughters’ fertility and offspring survival. Post-menopausal women help their daughters and nieces to take care of the offspring favouring the nutritional welfare of weaned children, especially when their mother is pregnant again or invested in a task outside the living place. This trans-generational transfer would induce an advantage to reduced senescence, and therefore favour the evolutionary selection of this trait. Grandmothering could favour biological functions related to longevity either through mutation-accumulation or the antagonistic pleiotropy strategies. In the former case, the selection against late deleterious variants is supposed to increase thanks to the contribution of gene pool from long-lived females that increased the reproductive success of their offspring. In the latter, grandmothering would favour mutations that increase adaptive performance at later ages, since it confers fitness benefits through increased alloparental care and reducing, at the same time, reproductive conflict (fertility/senescence trade-off). Empirical evidences of this fertility/senescence trade-off have been found in many studies [41–44] that highlight the role of grandmothering in the evolution of female post-menopausal longevity.

#### Intergenerational transfers model

In 2003, Lee proposed a biodemographic theory [39], where the use of the social dimension as a mathematical parameter succeeded to explain the peculiar life-history traits that characterize our species (lower fertility, longer life, increased investments in offspring). This theory generalizes and formalizes the arguments included in the “grandmother hypothesis” by integrating the classical selection due to fertility with the selection due to “intergenerational transfers” across the course of life. The term “intergenerational transfers” refers to the investment of resources in the offspring and other relatives, including parental care and support provided by others, such as grandparents and older siblings. Furthermore, the importance of intergenerational transfers in shaping the ageing patterns has been evidenced also for other social animals [45]. Unlike the classic theories of ageing [15, 16, 38], which claim that natural selection acts weakly at older ages, the model proposed by Lee suggests that selection continues also at older ages since it depends on the investments needed to produce a survivor at a given age. Therefore, selection could also act at post-reproductive ages if it leads to the survival and reproductive success of the juveniles.

### Life history theory

Another theoretical framework that includes the social dimension of ageing in complex ecological contexts is the “life history theory” [14, 46–49]. This theory explores how organisms, including humans, allocate their limited resources (such as energy, nutrients, and metabolic processes) across various life-history functions, including reproduction, maintenance, and longevity. The theory starts with the premise that resources are limited. These resources are needed for various life functions, including reproduction, maintenance (e.g. tissue repair, immune function), and somatic survival (longevity). During life, organisms make trade-offs in how they allocate these limited resources. Briefly, the theory suggests that resources allocated to one function (e.g. reproduction) come at the expense of other functions (e.g. maintenance and longevity). For instance, individuals with multiple children may experience a decrease in lifespan due to the energetic costs of pregnancy and childcare [50]. In humans, various cultural, societal, and environmental factors significantly influence personal choices and actions, which can in turn impact the allocation of resources during the life-course.

### Gene-culture coevolution models

Biological anthropology and the study of human evolution provide interesting insight into the complex interdependence between sociality and longevity. Gene-culture coevolution models suggest that social interactions play a crucial role in transmitting cultural and technological knowledge from one generation to the next [51]. According to Gintis (2011), two conditions are necessary for cultural transmission: a brain structure related to social behaviour and an extended lifespan to acquire and transmit cultural knowledge. In this regard, some studies argue that the evolution of genetics of lifespan follows a gene-culture co-evolutionary model with autocatalytic features briefly described below [52, 53]:

i. cultural practices create selective pressure for genetic variants that are associated with longer lifespan and slower ageing;
ii. as a consequence, the increase in lifespan leads the elders to have more time available to acquire knowledge and resources to pass on their offspring;
iii. this promotes further cultural progress, which, in turn, exerts selective pressure on genetic variants that favours a longer lifespan, creating positive feedback.

Studies on great apes support this model [54] and suggest that since during the learning phase the productivity is low, the acquisition of cultural knowledge can happen as long as there are intergenerational transfers from adults to juveniles.

### Embodied capital theory

The embodied capital theory (ECT) [55–57] originated in economics and sociology. It refers to the idea that individuals acquire and accumulate various skills, knowledge, and abilities over their lifetime, which are considered forms of “capital”. This capital, often referred to as “human capital”, represents an individual’s economic value and productivity [56, 58]. This theory has been applied to the study of hunter-gatherer ecologies, and in particular on the knowledge of high-level skills requested to forage high-quality and difficult-to-acquire foods [56], such as meat [59], tubers [60], and honey [61]. Indeed, according to this theory, the investment in learning extensive forageing techniques generated a return in the form of a dietary shift that, in turn, contributed to the enlargement of human brain capacity and to the evolution of slower human life history traits, such as delayed maturity, and extended lifespan [62].

### Exposome and allostatic load

The concepts of the exposome and allostatic load, as highlighted by Shiels and Stenvinkel, provide a modern theoretical framework to understand how sociality influences longevity [63, 64]. The exposome refers to the full range of environmental exposures throughout an individual’s life, including social factors, nutrition, and the physical environment [63]. Allostatic load reflects the cumulative physiological burden caused by chronic exposure to these stressors, particularly social and environmental challenges [64]. These concepts offer a valuable lens for examining how social structures and interactions shape biological ageing by affecting stress resilience and overall health.

Shiels and Stenvinkel emphasize that chronic stress and socio-environmental pressures, key aspects of the exposome, lead to an accumulation of allostatic load, which accelerates ageing by modulating cellular processes such as inflammation and oxidative stress [63, 64]. In this context, sociality plays a crucial role in mitigating these stressors. Strong social bonds and supportive environments help reduce allostatic load by buffering individuals from chronic stress, thereby protecting against the oxidative stress that drive biological ageing. As a result, these social interactions can slow down ageing and promote longevity; conversely, social isolation and adverse environments exacerbate oxidative stress and allostatic load, accelerating ageing and increasing the risk of age-related diseases such as cardiovascular disease and cancer [65].

## Bioanthropological evidence linking sociality and natural variation in lifespan

In this section, we will show how social dynamics, and in particular intergenerational transfers and resource sharing, may have contributed to the evolution of lifespan variability using evidence from biological anthropology. In this perspective, studying the evolutionary dynamics that connect sociality and longevity and their patterns of natural variations is essential to unveil the multiple dimensions of the contemporary ageing society and to address the ongoing demographic shift.

**Fig. 2:**
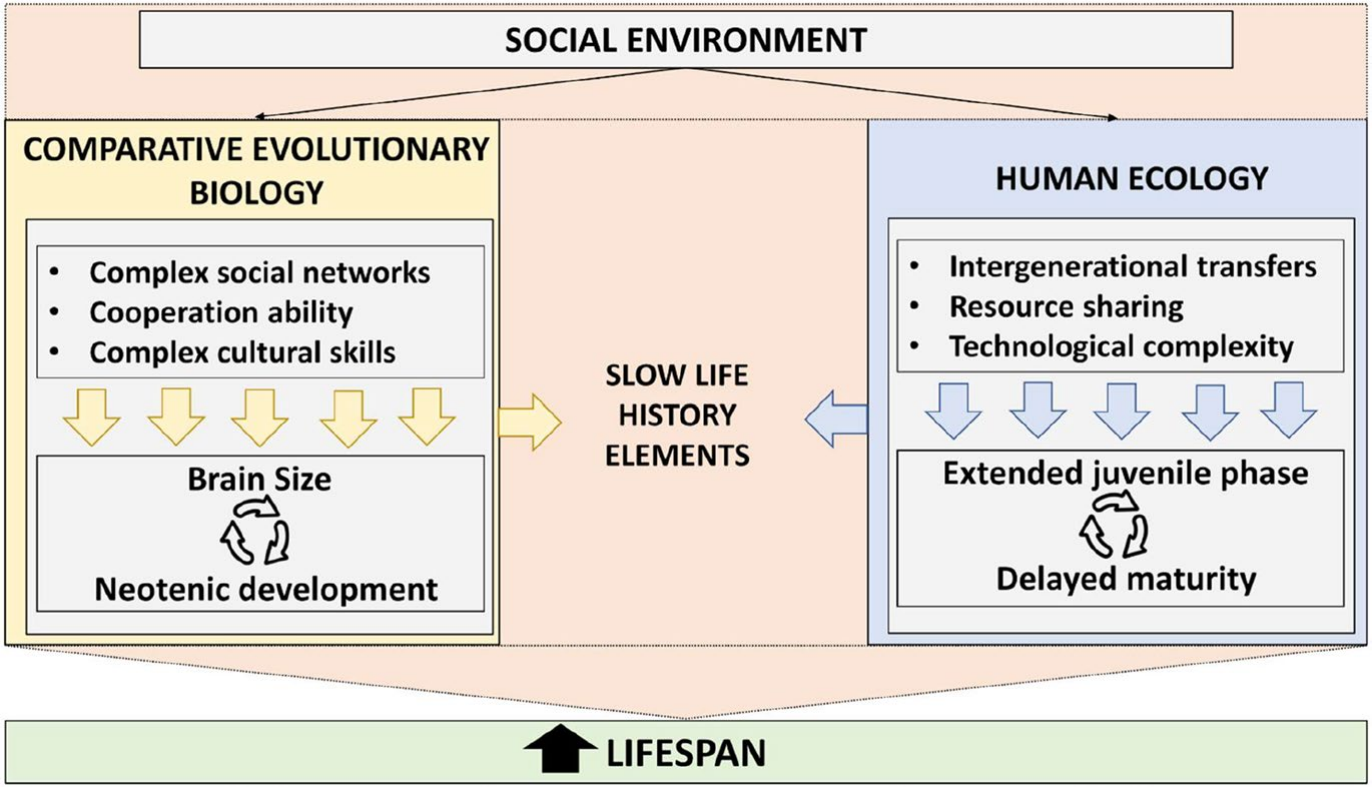
Link between social environment and extended lifespan in pre-industrial society. The lens of comparative evolutionary biology provides evidences of the social determinants that favours biological mechanisms (in particular the evolution of neoteny and larger brains) responsible for the differences in extended lifespan between modern human hunter-gatherers and other primates. The lens of the human ecology provides evidences of the social determinants that favours biological mechanisms (in particular the delayed maturity and the extended juvenile phase) responsible for the differences observed among human groups living in different ecological niches

In this chapter, we first summarize data showing how evolutionary-conserved social dynamics, such as cooperation ability in complex social interactions, contributed to the extension of the lifespan in humans with respect to other primates (section “Insights from comparative evolutionary biology” and Fig. 2 left). We then offer a brief overview on how such evolutionary-conserved dynamics are embedded in other social animal. Next, through studies in human ecology and biodemography, we point how sociality contributes to natural variation in lifespan among pre-industrial human groups, making abstraction of the confounding factors present in post-industrialized populations (such as medical practices). In particular, we will discuss how slow life history elements (such as the extended lifespan) are influenced by intergenerational transfers and resource sharing in ecological niches with higher technological complexity (see section “Insights from human ecology and pre-industrial societies” and Fig. 2 right). In conclusion, we will describe the link between sociality and natural variations in lifespan in post-industrial society (section “Insight from post-industrial societies”).

### Insights from comparative evolutionary biology

In humans, technical progress leads to an increase in life expectancy and a substantial decrease in mortality rates, especially in comparison to wild chimpanzees [66]. In fact, although the time required for the mortality rate to double is quite similar between modern human hunter-gatherers and chimpanzees [67], the percentage of surviving individuals at adult ages is much greater in the former, with an additional lifespan at age 45 about three times higher in human hunter-gatherers than in chimpanzees [68].

These observation has been explained by the fact that neoteny, marked in humans by immaturity at birth and slow postnatal maturation, extends lifespan [69]. It is hypothesized that the evolution of neoteny and longer developmental period (that in turn contribute to a longer lifespan) plays a role in brain size, structure, and connectivity typical of *Homo sapiens*. Some authors linked these changes to the specific social environment of *Homo sapiens*, for example human ontogeny occurs within the context of a highly cooperative social group whose members collaborate and interact [70]. The comparison between humans and great apes showed that human children learn more information and cultural skills via teaching from others; this has been observed in societies of all types [71]. Moreover, human cultural organization in all societies is characterized by shared information between generations leading to cumulate cultural artefacts, skills, and technological knowledge.

A recent paper of Richerson and Boyd [72] presents important insights on this topic, providing evidence showing that the long, slow human life history coevolved with our large brain. Brain growth is closely linked to the high complexity of human social networks that in turn are crucial for the transmission of cultural knowledge. In this regard, it is suggested that by the late Pleistocene the diffusion of the hunting/extractive niche led to selection for larger brains and to the need for cooperative breeding to provision such brains. Thus, humans became highly dependent on complex cultural skills for subsistence and on rules to organize a complex social life. The dependency from cultural traditions and complex social networks transformed human life history elements, leading to an extended juvenile period (to learn subsistence and social skills), post-reproductive survival (to help conserve and transmit skills), and in turn, to an extended lifespan. Bioarchaeology seems to converge on similar findings: a dramatic increase in the number of adult individuals in anatomically modern humans was observed in the Early Upper Paleolithic, a process that has been linked to population expansion and cultural innovations of that period [73], likely placing the relation between social factors and human ageing way back in time. Molecular data support that the evolution of neoteny would be responsible for the difference observed in the extension of lifespan between chimpanzees and individuals of pre-industrial societies [74, 75]. The vast majority of miRNA and gene expression changes in the prefrontal cortex of humans and rhesus macaques over the species’ lifespan represent reversals, or extensions, of developmental patterns [76], suggesting a link between developmental regulation and expression changes during ageing.

#### Comparative insights from other social animals

The naked mole rat (*Heterocephalus glaber*) offers an intriguing example of how sociality may contribute to exceptional longevity in different species. These small, burrowing rodents are eusocial, living in large colonies with a complex social structure similar to that of some insect species, such as ants and bees [77, 78]. The colonies are typically comprised of a single breeding female, the queen, a few breeding males, and numerous non-reproductive workers. Naked mole rats are remarkable for their longevity, with lifespans that can exceed 30 years—far longer than other rodents of similar size [79, 80]. This extended lifespan is associated with several unique physiological traits, many of which are believed to be influenced by their social structure [69, 79]. The eusocial structure of naked mole rat colonies ensures a division of labour that promotes colony efficiency and survival. Non-reproductive workers undertake various tasks, including foraging and caring for the queen’s offspring, which enhances the overall fitness and longevity of the colony [69, 81]. The social hierarchy and interactions within the colony may influence hormonal pathways that contribute to longevity [82]. For example, the queen’s presence can affect the physiology and behaviour of other colony members, potentially enhancing their stress resistance and health [81].

Interestingly, the social hierarchy within naked mole rat colonies also introduces complexities in how stress impacts longevity [80, 83, 84]. Previous studies suggest that while the queen experiences reduced stress, subordinate individuals often face social stress due to frequent bullying [84]. This raises the question of whether subordinates, who endure chronic stress, experience reduced lifespans. Incorporating this perspective adds nuance to the understanding of how social structures, stress, and longevity interact.

The interaction between social structure and biological resilience in naked mole rats can be further understood through the lens of the exposome and allostatic load (see section “Exposome and allostatic load” for details) [63]. In naked mole rats, social cohesion and division of labour reduce allostatic load, thereby mitigating chronic stress and its detrimental effects on health [63, 64]. This reduction in allostatic load through social structures is complemented by underlying molecular mechanisms that further enhance the naked mole rat’s resilience to ageing. At the molecular level, naked mole rats exhibit elevated activity of the Nrf2 gene, which regulates cellular defences against oxidative stress and inflammation, contributing to their resistance to age-related diseases such as cancer and cardiovascular conditions [63].

Further strengthening the case for the unique sociality of naked mole rats, previous studies on the vocal communication (“language”) in these animals show how their advanced social interactions may also contribute to their longevity [85]. Additionally, recent findings on prolonged pedomorphosis in naked mole rats, manifesting in sustained neurogenesis and parasympathetic-driven cardiac function, provide further evidence of the physiological benefits conferred by their eusocial structures [86].

Comparative insights from other social animals, such as elephants and dolphins, further illustrate how social structures and behaviours contribute to extended lifespans [87]. Elephants, for instance, live in matriarchal societies where older females play crucial roles in guiding and protecting the herd [87]. The matriarchs’ extensive knowledge of their environment, such as the locations of water sources and safe migratory routes, is vital for the survival of the group. Their social bonds and cooperative behaviour, including alloparental care where individuals assist in the care of offspring that are not their own, contribute to the overall health and longevity of the herd. Dolphins also demonstrate the benefits of complex social structures [88]. They live in fluid, multi-level societies where cooperation, social learning, and strong social bonds are common [88]. Dolphins engage in cooperative hunting, share knowledge about feeding strategies, and provide care for sick or injured members [88]. These behaviours not only enhance individual survival but also improve the group’s overall resilience and adaptability, leading to longer lifespans. These examples from elephants and dolphins, alongside the naked mole rat, highlight common evolutionary strategies where sociality and cooperative behaviours play pivotal roles in promoting longevity. In all these species, social structures facilitate resource sharing, collective defence against predators, and efficient care for the young and vulnerable, thereby enhancing survival rates and extending lifespans. These insights underscore the potential for sociality to drive longevity across diverse taxa, offering a broader understanding of the interplay between social behaviour and ageing. By examining these diverse species, we can better understand the evolutionary advantages of sociality and its impact on lifespan. The mechanisms observed in naked mole rats, elephants, and dolphins provide valuable models for exploring how social structures and behaviours may influence human ageing and longevity, emphasizing the importance of fostering social connections and support networks in promoting healthy ageing.

While many social species display exceptional longevity, there are notable exceptions that challenge the straightforward correlation between sociality and lifespan. For instance, antelope species, which live in herds, have relatively short lifespans despite their social organization [89]. Similarly, solitary species like the *Spalacidae* mole rats exhibit remarkable longevity despite their lack of complex social structures [89]. These examples highlight that factors such as predator pressure, metabolic demands, and ecological niches may play crucial roles in determining lifespan, independent of social behaviours. This suggests that while sociality is a significant factor, it is not the sole determinant of longevity and must be considered alongside other evolutionary and ecological pressures.

### Insights from human ecology and pre-industrial societies

Studies in human ecology and observations of pre-industrial societies provide evidence about the role of sociality, in the form of intergenerational transfers of food, knowledge, and skills, on certain life-history traits, such as delayed maturity, extended juvenile phase, and in turn extended lifespan [40, 56, 90, 91]. However, how the social environment shapes human lifespan extension depends mainly on the technical complexity of a given niche, related in particular to the required skills and knowledge (such as the hunting method or the forageing complexity). In fact, according to [74], technical progress may be responsible for the great differences in the mortality risks of individuals living in different ecologies. Recently, the role of intergenerational transfers in shaping extended lifespan in high-skill forageing niches has been described considering eight different populations of contemporary human hunter-gatherers and horticulturalists [92]. The authors used a demographic approach and combing data on the caloric production and demand with survival and fertility data. They noted that skills-intensive forageing ecology, where late adult independence and late peak production are observed, select for certain slower human life-history traits through positive feedback between longevity and late-life transfers. In particular, they showed that intergenerational transfers of production surpluses from adults to juveniles favour the extension of the learning phase and the delayed maturity [92]. These observations were recently supported by another study on 714 children and adolescents from 28 pre-industrial societies [93]. This study showed that forageing returns increase slowly in skills-intensive forageing ecological niches, where resources (tubers and game) are more difficult to extract, and thus the peak production is observed late in adulthood. On the other hand, forageing returns increase rapidly during childhood for easier-to-extract resources (fruit and fish/shellfish), and thus the peak of productivity is reached by adolescence [93]. Since the lower productivity during the learning phase is offset by a higher productivity at adult ages, the time spent in the acquisition of high-level skills and knowledge drives the selection of lower adult mortality rates and extended lifespan, because the return on investment in learning occurs at older ages, when the productivity is higher [55, 92].

The study of family structure and features in different human populations is also crucial for understanding the complex relation between sociality and ageing and influence intergenerational transfer. For example, family structures in pre-industrial societies play a pivotal role in shaping intergenerational transfers and supporting sociality. Extended kin networks, including grandparents and other relatives, provide essential support in child-rearing and resource sharing [94]. These kin networks not only enhance survival rates by pooling resources but also foster pro-social behaviour and cultural transmission among family members, contributing to the overall health and longevity of the community.

Thus, the intricate interplay of family structure, social dynamics, and resource sharing highlights the critical role that sociality plays in shaping health and longevity outcomes in traditional societies.

### Insight from post-industrial societies

In post-industrial societies, the concept of “intergenerational transfers” is much more complex because it is not limited only to the transfers of food, knowledge, and skills that we observe in pre-industrial societies, but it includes also different kind of transfers, such as those that involve public programmes at different life-cycle stages, as well as all private familial transfers. Nevertheless, it is well established that prosocial behaviour and social integration have been positively associated with health and reduced mortality in many post-industrial societies [3, 95]. In the last decades, death rates of aged populations have sharply declined thanks to a well-developed medical system [96], but most probably, thanks also to factors including, between many others, the level of social support present in these societies [97].

The notion that the level and the quality of social support have a direct effect on health and mortality became popular thanks to two ground-breaking reviews published in 1976 [98, 99]. Both studies argued that social support, particularly from closely related individuals, could protect from the physiological and psychological consequences of exposure to stressful situations improving the resistance to pathogens or other heath menaces. In 1979, a study performed on a random cohort of about 7000 adults from Alameda County (CA, USA) which were followed for at least 9 years demonstrated that individuals with higher social and community relations have a lower probability of death than those with few social interactions, independently from other lifestyle or medical factors [100].

A plethora of studies and meta-analyses, summarized by Jaime Vila in 2021 [97], have provided experimental ground for the role of social relations and intergenerational transfers in determining the health status and longevity of individuals in an industrial society.

Recently, a study on 34 post-industrialized countries, using the data from NTA project (https://ntaccounts.org/), demonstrated that these transfers (both within families and within communities) are negatively related to national mortality and positively related to longevity, suggesting (1) a beneficial effect of pro-social behaviour on population longevity [11] and (2) that this effect is present at different life stages [101].

Cultural differences and variations in family structure across human populations significantly influence the relationship between sociality and longevity. A clear example of that is seen in the Chinese social norm of preference for sons. In China, the cultural preference for sons profoundly shapes the social support structures available to older adults, particularly in rural areas where traditional patriarchal and patrilineal norms remain influential. Recent research highlights that rural women who live with their sons benefit from stronger support networks, especially from non-relative friends [102]. This support is significantly enhanced by the presence of sons, as they are culturally seen as the primary caregivers and providers of old-age security. In contrast, this pattern does not extend to rural women living with daughters, reflecting the deep-rooted cultural expectations that prioritize sons in the provision of care and social resources. Such findings underscore the gendered nature of support systems in rural Chinese communities, where sons, more than daughters, facilitate broader social engagement and contribute to the well-being and ageing process of older adults [102].

Similarly, the ways in which cultural norms shape intergenerational support and social interactions vary significantly across different cultural contexts. For instance, in many Asian cultures, the expectation of filial piety drives children to provide both financial and emotional support to their ageing parents. This expectation reinforces family ties favouring care practices that significantly enhance the well-being of older adults and foster longevity [103]. In contrast, Northern European societies, where comprehensive welfare systems provide substantial support for the elderly, exhibit different patterns of intergenerational transfers [104]. However, while these societies demonstrate lower rates of upward financial transfers from children to parents, the emotional support provided by family remains crucial for mental health and resilience for the elderly [105, 106]. Southern European countries often maintain strong family cohesion, where intergenerational transfers are characterized by frequent support from adult children to their parents. This cultural emphasis on familial obligations fosters close-knit relationships, enhancing both social support and health outcomes for older adults. In these societies, grandparents are often actively involved in childcare, contributing to a robust intergenerational exchange that benefits the family unit as a whole [105, 106].

The sum of these findings confirms a direct correlation between the degree of social interactions and reduced risk of disease and mortality, where resource sharing and intergenerational transfers play a major role. Furthermore, the interplay of family structure, kinship ties, and social support is essential for understanding how sociality contributes to health outcomes in post-industrial societies.

#### Blue Zones: socio-cultural determinants of longevity

“Blue Zones” are regions around the world identified as having populations with significantly higher-than-average lifespans [107–109]. Blue Zones include five key regions: Okinawa (Japan), Sardinia (Italy), Nicoya Peninsula (Costa Rica), Ikaria (Greece), and the Seventh-day Adventist community in Loma Linda (CA, USA) [109]. These areas share unique socio-cultural and environmental traits that contribute to their inhabitants’ exceptional longevity [109]. Individuals living in Blue Zones have been observed to exhibit a biological age younger than their chronological age [110]. This region-specific deceleration of ageing processes in inhabitants of Blue Zones and other similar longevity regions is attributed to the distinctive lifestyle and social factors deeply embedded in their cultures, which promote both physical and social health [109, 111, 112]. These practices include strong social networks.

In Blue Zones, maintaining close-knit communities and strong family ties is a fundamental aspect of daily life [111, 113–116]. For instance, Okinawans practice “moai”, a tradition of forming lifelong social networks that provide financial and emotional support [117]. Similarly, Sardinians maintain robust familial bonds and social cohesion, which fosters a sense of belonging and security [118]. Many Blue Zone cultures emphasize the importance of intergenerational living, where multiple generations reside together or in close proximity [109, 118]. This living arrangement promotes the exchange of knowledge, wisdom, and care across generations, enhancing the well-being of both younger and older members. When elder family members play active roles in the upbringing of children, it reinforces family bonds and provides a sense of purpose for the elderly. Strong social networks play a crucial role in cultivating a sense of purpose, a key insight from Blue Zones research. In regions like Okinawa and Nicoya, this sense of purpose is known as “ikigai” [119, 120] and “plan de vida” [109], respectively. A well-defined purpose in life greatly enhances psychological resilience and competence, promoting motivation, determination, and a positive outlook [121]. These robust social networks provide emotional support, social engagement, and opportunities for meaningful roles within the community, enhancing individuals’ feelings of connectedness and belonging. This interconnectedness helps to reduce stress and anxiety, protecting against the harmful effects of chronic stress on biological ageing processes [122, 123].

The social structures and cultural practices in Blue Zones exemplify the positive impact of sociality on ageing and longevity. The strong social networks, intergenerational interactions, and communal support systems provide emotional stability, reduce stress, and promote mental health. These social factors are crucial in mitigating the detrimental effects of social isolation and loneliness, which are significant risk factors for morbidity and mortality in ageing populations. Understanding the socio-cultural and environmental factors that contribute to the longevity of Blue Zone populations offers valuable insights for ageing societies worldwide. Promoting social connections, fostering intergenerational relationships, encouraging activities that result in stress reduction can collectively enhance the healthspan and lifespan of individuals. The integration of these principles into public health policies and community planning can help address the challenges of an ageing population, fostering environments that support longevity and well-being.

The fact that supportive social relationships are important for well-being and longevity suggests that understanding the neurological and biological factors underlying this phenomenon could pave new avenues to improve quality and duration of life [124].

## Physiological mechanisms linking sociality and lifespan

**Fig. 3:**
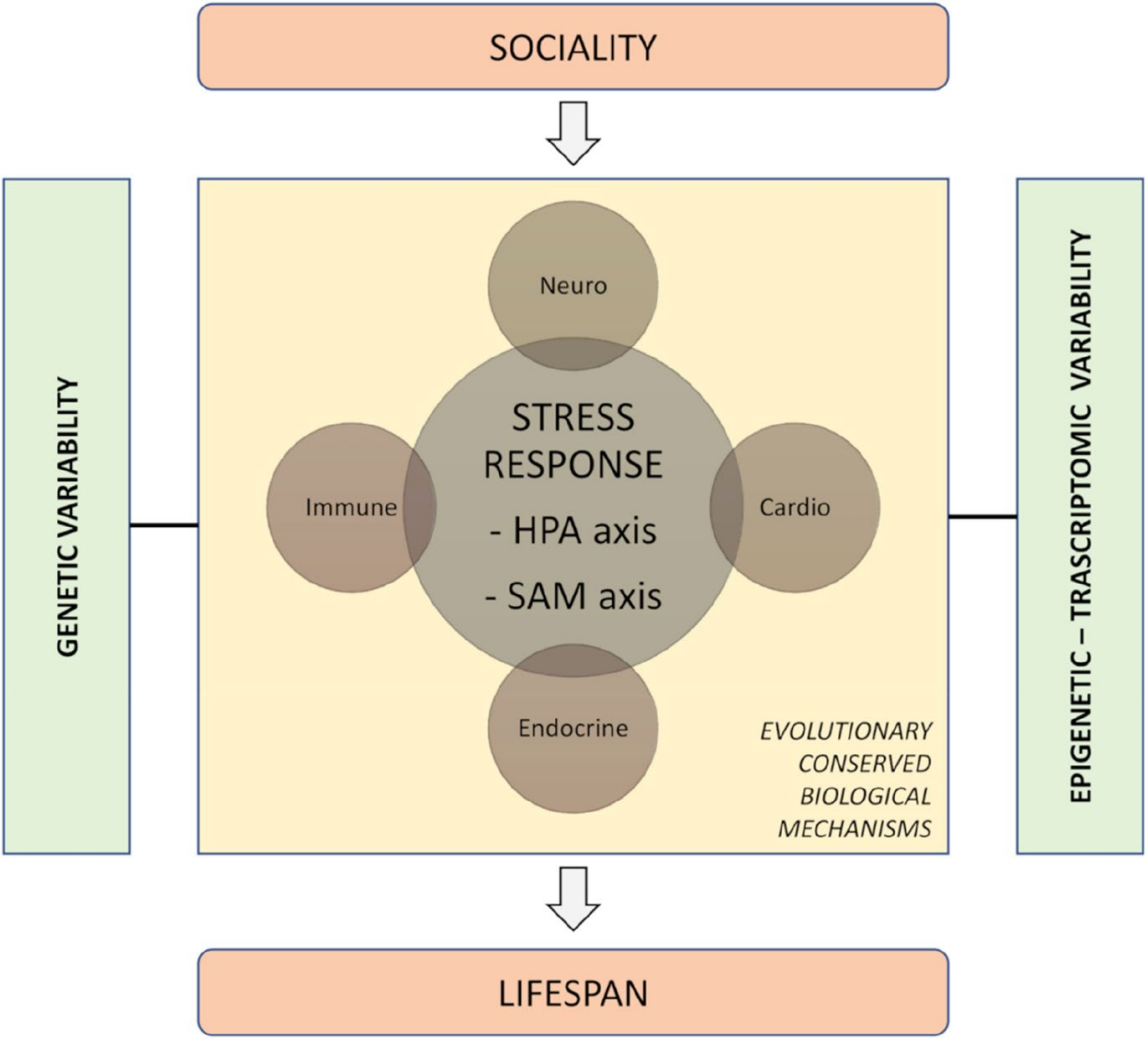
Social dynamics impacts on evolutionary conserved mechanisms of stress response that involved HPA (hypothalamic–pituitary–adrenal) and SAM (sympathetic-adrenomedullary) axes. The effect of sociality—in reason of the systemic nature of stress response—has been observed in immune, neuroendocrine, and cardiovascular systems. These biological changes that could include both genetic and epigenetic changes can ultimately influence lifespan

As mentioned above, the impact of social environment on health and lifespan is a conserved feature of human populations, and several mammalians orders [10, 34] suggesting the involvement of shared evolutionary-conserved physiological processes (Fig. 3). The social environment has been identified both as a source of relief and one of the primary sources of challenging stimuli that can induce a stress response [125]. “Stress” is the process through which an organism reacts to environmental stimuli that challenge the stability of either its physiological conditions (homeostasis) or of its mental/psychological landscape, whereas “stress response” is the activation of key physiological or neuropsychological pathways aimed to maintain organism stability and survival in a challenging environmental context.

Chronic socially induced stress exposure in humans and animal models accelerates the progression of metabolic, cardiovascular, and neurological diseases as well as malignancies [126] and is a predictor of several mental and physical health conditions including depression, schizophrenia, bipolar disorder, cardiovascular disease, autoimmune disorders, and impaired cognitive functions [127–134]. More generally, chronic stress is associated to earlier mortality [135], and experimental animal models exposed to stress present a shorten lifespan that can be alleviated by social interactions: according to the so-called stress-buffering hypothesis, “the availability of a conspecific reduces the activity of stress-mediating neurobiological systems” [136].

### Neurophysiological pathways of stress

The neurophysiological pathways involved in the activation of the response to stress implicate the hypothalamus and other brainstem areas involved in inducing specific defensive reactions (freezing, startle response, fight-or-flight) through activation of the autonomic nervous system and of the neuroendocrine system. It has been suggested that the chronic activation of these defensive pathways, also called the “default stress response” [137, 138], transforms their physiological effects from life-saving to self-harming favouring increase morbidity and mortality through deregulation of the cardiovascular, neuroendocrine, and immune systems.

Two neurophysiological signalling systems are involved in the activation of a stress response: the hypothalamic–pituitary–adrenal (HPA) axis and the sympathetic-adrenomedullary (SAM) system. Their activation results in the increases of circulating glucocorticoids (cortisol) and catecholamines (adrenaline) respectively which, both, are needed to prepare the body for a fight-or-flight response. An initial threatening sensory input activates cerebral cortical and subcortical structures including the amygdala and the hypothalamus, and finally induces autonomic, endocrine, and motor responses to protect the organism from the menace. The “stress-buffering hypothesis” suggests that social support has positive effects on health status and longevity through the reduction of the negative effects that prolonged activation of the HPA and SAM axes has on different physiological systems.

Interestingly, both these systems converge on the adrenal gland, which has been implicated in another important theory of sociability: the domestication syndrome. Seminal observations have proposed that the selection of breeders from generation to generation on the sole criterium of “docility”, an adrenal phenotype, induced the co-selection of hormonal, behavioural, and anatomical traits [139, 140]. In humans, some theories propose that an auto-domestication occurred during human evolution [141–143], favouring individuals with higher sociability.

Furthermore, experimental studies in humans have confirmed the importance of the HPA axis for the stress-buffering effect exerted by positive social input: indeed, exposure to attachment figures provokes cortisol level reduction at different periods of life [136, 144]. The involvement of the SAM signalling system on stress-buffering has been more difficult to demonstrate experimentally due in great part to methodological difficulties [97, 145, 146].

The link between socially induced stress and physiological mechanisms involved in different life-history traits has been demonstrated also in non-human experimental studies, suggesting that crucial biological mechanisms are involved. A study on red squirrels [147] showed that social “negative” cues have an intergenerational effect on the growth rate, and more precisely, that high-density cues, accomplished via playbacks of territorial vocalizations, led to increased offspring growth rates. A higher social-induced stress would increase glucocorticoid levels in mothers, which in turn affect the growth rate of the pups. However, offspring born from social stressed mothers have a reduced adult lifespan [148], suggesting that faster offspring growth might incur a cost to offspring later in life. Thus, these studies suggest an indirect link between sociality and lifespan, mediated by physiological and behavioural stress response of mothers and the resulting consequences on the offspring.

### Effects of social isolation and stress on the cardiovascular system

In his famous lecture at the Sorbonne in 1885, Claude Bernard discussed how the physiology of the heart was intimately connected to that of the brain and how cardiac activity was in part mediated through the vagus nerve [149]. Indeed, heart rate is regulated by the autonomic sympathetic and parasympathetic nervous systems [150]: the sympathetic nervous system increases heart rate, whereas the parasympathetic nervous system reduces it.

Prolonged stress results in imbalanced regulation of the heart with increased sympathetic and decreased parasympathetic stimulation, resulting in higher heart rate with increased morbidity and mortality [138]. A reciprocal interconnection between the brain and the heart is at the origin of the integration of cognitive, affective, and autonomic systems in a dynamic model of stress regulation suggesting that reduced stress would stimulate the prevalence of the parasympathetic system resulting in increased health and longevity [151, 152]. In a safe milieu and in a socially supportive situation, an increased parasympathetic control by the vagus nerve would slow the heart, reduce the activity of the HPA axis, and avoid fight-or-flight sympathetic response.

A recent study [153] has determined a direct association between social isolation, loneliness, and coronary heart disease and stroke mortality. According to this study, social isolation and loneliness are associated with about a 30% increased risk of heart attack or stroke, or death from either. Evidence is most compelling for an association between social isolation and death from heart disease and stroke, with a 29% increase in the risk of heart disease death, and a 32% increased risk of stroke and stroke death [154]. It seems plausible that chronic physiological and psychological responses to social isolation-stress might be at the origin of this increased risk of death suggesting that measures are taken to alleviate social isolation in our society.

### Endocrine effects on sociality and ageing

During ageing, the endocrine system becomes less efficient, resulting in a parallel decline of social and physiological capacities with increased morbidity and a higher risk of death. For example, in men, low testosterone levels lead to a progressive decline in muscle and bone mass with reduced physical performances and, in women, loss of oestrogens strongly increases the risk of osteoporosis and fracture.

The main hormones involved in the control of social behaviour are the two nonapeptides oxytocin (OT) and arginine-vasopressin (AVP), and the gonadal hormones testosterone and oestrogens. These hormones mutually regulate their secretion and converge on the regulation of target organs through complex interacting pathways. OT and AVP are among the earliest neurohormones appeared during evolution [155], they are produced by magnocellular neurons in the hypothalamus and are then secreted in the blood stream and act throughout the brain [156, 157]. Centrally, these hormones target brain regions involved in social cognition including the amygdala, the striatum, and the hippocampus [158].

OT is a neuropeptide produced by the paraventricular nucleus of the hypothalamus and released into the circulation by the posterior pituitary. OT release in the brain is involved in the formation of mother–offspring bonds [159]. It is also involved in the regulation of the HPA axis, both in response to a stressing situation or to the presence of social support [160, 161], resulting in the activation of pro-social protective behaviours.

AVP has a chemical structure closely related to that of OT [162]. Besides its fundamental physiological functions in the control of blood pressure and kidney function, AVP has been shown to play an important role in determining the social behaviour of many vertebrate species [163–168].

In mammals, AVP is produced by several hypothalamic regions including the paraventricular (PVN), supraoptic (SON), suprachiasmatic nuclei (SCN), the nucleus circularis (NC), and the preoptic area (POA). Furthermore, AVP is produced by specific sensory regions of the CNS including the olfactory bulb (OB) and by the amygdala where its expression is steroid-dependent and sex-specific [169]. The effects of AVP and OT on social behaviours are due to their ability to activate brain regions involved in social recognition [170], maternal care, pair bonding, communication [171], aggression, cognition [172], and stress and anxiety-like behaviours [173, 174].

Various studies have shown that OT, AVP, and gonadal hormones interact to determine social recognition and social behaviours [175]. For example, oestrogen enables social recognition by regulating hypothalamic OT production and its receptors in the medial amygdala. In male rodents, AVP stimulates social recognition by acting on the lateral septum. In general, it appears that the fine regulation exerted by gonadal hormones on OT and AVP can adjust social behaviours in a sex-specific manner, reinforcing the notion of the central role of these neuroendocrine systems in the regulation of the sex-specific behaviours. These behavioural effects do not only impinge on reproduction, but affect more globally the insertion of individuals in a social structure. During ageing, the progressive reduction of gonadal hormones both in males and females reorients social behavioural patterns, adapting them to the different epochs of life.

### Immunity and inflammation and the relation between sociality and lifespan

The major role of the immune system is to protect the organism from external pathogens through the process known as inflammation. The inflammatory reaction is activated by small secreted proteins known as pro-inflammatory cytokines which include, for example, IL-1, IL-6, and TNF-α. These cytokines are released predominantly by Th1 cells, CD4 + cells, macrophages, and dendritic cells. They play a central role in regulating proliferation, activation, differentiation, and homing of immune cells to the sites of infection where they can fight pathogens, including viruses (see, for example [176],). In addition to immune cells, several other organs and tissues also release inflammatory cytokines. Adipocytes can secrete pro-inflammatory cytokines such as IL-6 and TNF-α, especially in the context of obesity [129, 177–181]. This chronic low-grade inflammation in adipose tissue is a significant contributor to metabolic disorders and age-related heightened state of systemic inflammation. Endothelial cells lining the blood vessels can also produce cytokines like IL-1 and IL-6 [182]. Age-related endothelial inflammation plays a key role in the development of atherosclerosis and other cardiovascular diseases as well as vascular cognitive impairment and neurodegenerative diseases [183, 184].

Beside their action on the immune system, pro-inflammatory cytokines also affect the activity of the brain and can influence social behaviour increasing sensitivity to both negative and positive social experiences, which results in variations of the likelihood to develop diseases or face pathogens [185, 186]. Furthermore, negative social experiences such as parental separation, mourning, isolation, and loneliness influence directly the immune system by increasing pro-inflammatory cytokines [187]. A large meta-analysis, based on 41 independent studies, confirmed these findings showing a significant correlation between low social support and inflammation [188]. Chronic inflammation associated with low social integration and/or social support can affect negatively the risk of many common pathologies including cancer, diabetes, cardiovascular disease, kidney disease, neurodegenerative disorders, non-alcoholic liver disease, and autoimmune diseases, causing higher morbidity and mortality [189].

Moreover, Yang and colleagues [101] provided evidence on the role of social connections on reducing the risk of inflammation and hypertension in adolescence, young, and late adulthood, suggesting that the impacts of social relationships on physiological mechanisms emerge in adolescence and midlife, but persist also into old age. From this point of view, the impact of sociality on inflammation could be seen as an effect on the rate of ageing, contributing to the process known as “inflammageing” [13]: the sterile, low-grade inflammation that develops while growing older and contributes to the pathogenesis of age-related diseases. The process of inflammaging is driven by multiple factors, including the accumulation of senescent cells, which secrete pro-inflammatory cytokines, chemokines, and other factors collectively known as the senescence-associated secretory phenotype (SASP) [190, 191]. These secreted factors contribute to chronic, low-grade inflammation and can affect the surrounding tissue environment. Chronic inflammation from SASP can exacerbate tissue damage and contribute to the development of age-related diseases. Inflammaging is characterized by an increase in circulating pro-inflammatory cytokines and is associated with a higher risk of many age-related diseases, such as cardiovascular disease, type 2 diabetes, neurodegenerative diseases, and certain cancers. This chronic inflammatory state stimulates the senescence of the immune system reducing its capacity to eliminate senescent cells and inflammatory factors, creating a self-sustaining cycle of inflammation and senescence. In this sense, inflammation has been recognized as one of the hallmarks of ageing, and its reduction could be a potential strategy for anti-ageing intervention [192]. Low social integration is, from this perspective, one of the many stimuli which contribute to the inflammageing process determining the evolution of morbidity and longevity [192, 193]. Indeed, many studies have confirmed that social stressors are particularly strong triggers of inflammation [194–196].

It has been shown that a feedback regulatory loop exists between the function of the immune system and socio-behavioural conditions. Indeed, on one side, psychoneuroimmunology has shown that the quantity and quality of social connections can directly affect immune responses, while, on the contrary, pathological conditions can transiently affect social interactions as it has been seen, for example, during the COVID-19 pandemic. “Social immunology” is a research approach, which integrates socio-economic, political, and environmental factors with the effects of pathogen exposure on the immune responses. Social immunology is therefore a “holistic understanding of the effects of social contexts on the patterning of morbidity and mortality” highlighting risk factors related to impaired immune function [197, 198]. Understanding the complex interplay between sociality, immunity, and inflammation can inform strategies to improve health and longevity. Interventions aimed at enhancing social support and reducing social isolation could mitigate the inflammatory processes associated with ageing and chronic diseases, thereby promoting healthier ageing and extended lifespan.

## Genetic and epigenetic mechanisms linking sociality and lifespan

The association between lifespan and social behaviours has very profound roots. This observation implies the presence of significant genetic and epigenetic factors that are shaped by social dynamics and, in turn, play a role in the natural variations observed in lifespan.

Understanding these genetic and epigenetic determinants is an entry point to decipher the impact of sociality on the physiological mechanisms which determine human ageing.

### Genetics

The fact that social behaviour in many mammalian species, including humans, is associated with lifespan suggests that some evolutionary conserved and highly pleiotropic genetic mechanisms might be involved.

An interesting example is given by the targeted inactivation of *Dlx5* and *Dlx6* which encode two homeobox transcription factors expressed by developing and mature GABAergic interneurons in the forebrain. Mice in which *Dlx5* and *Dlx6* are simultaneously inactivated selectively in GABAergic interneurons present a hyper-vocalization and hyper-socialization phenotype and behavioural patterns suggesting reduction of both anxiety-like behaviour and obsessive–compulsive activities [199]. These animals present also a 25% body weight reduction associated with a marked decline in white and brown adipose tissue. Remarkably, both inactivation of *Dlx5/6* in GABAergic neurons results in a 33% longer median survival and hallmarks of biological ageing are all improved in these mutant animals. These data imply that GABAergic interneurons can at the same time regulate social behaviour and lifespan through *Dlx5/6-*dependent mechanisms. Interestingly, comparison of the *DLX5/6* genomic regions from Neanderthal and modern humans has permitted to identify an introgressed Neanderthal haplotype (*DLX5/6*-N-Haplotype) present in 12.6% of European individuals that covers *DLX5/6* coding and regulatory sequences. The *DLX5/6*-N-Haplotype is not significantly associated to “autism spectrum disorders” but includes the binding site for GTF2I, a gene associated to Williams-Beuren syndrome, a neurodevelopmental disorder characterized by hyper-sociability and hyper-vocalization. Interestingly, the *DLX5/6*-N-Haplotype is significantly underrepresented in semi-supercentenarians (> 105 years of age), a well-established human model of healthy ageing and longevity, suggesting its potential involvement in the co-evolution of longevity, sociability, and speech [200].

An experimental evidence comes from the study of Schwarz and colleagues [201] based on the genetics of neurodegenerative disease. The authors provide some evidence that alleles located in CD33 gene that protect against age-related cognitive deterioration could result from kin selection late in life through increased survival of younger kin. Extended lifespan is certainly an even more complex phenotype where neurological health is one of the most important factors. The authors support the notion that selection by inclusive fitness may be strong enough to favour alleles protecting from cognitive decline, a condition that would have compromised the emergence of cumulative culture.

### Epigenetics and gene regulation

Epigenetic changes are among the most important molecular “hallmarks of ageing” [202] and are associated to the decline in cellular functions seen in ageing and age-related conditions. During ageing, epigenetic changes can lead to aberrant gene expression, reactivation of transposable elements, and genomic instability [203]. DNA methylation (DNAm), one of the most important epigenetic modifications observed during ageing, mediates the effect of the early-social environment on later-life phenotypes [204–206]. A recent study on a wild population of spotted hyenas [207] found that more maternal care and social connectedness during the later subadult life stage, after leaving the den, are associated with higher global (%CCGG) DNAm, a marker of genomic instability and overall health. This same study has also identified differential DNAm in five genes related to inflammation, immune response, and ageing that may connect maternal care and the manifestation of stress-related phenotypes later in life.

The importance of DNAm as the process at the interface between the early social experiences and later-life phenotypes extends also to our own species. A recent study [208], using methylation data of 494 participants (age 20–22 years) recruited from a longitudinal birth cohort survey in the Philippines, showed that nutritional, microbial, and psycho-social exposures in infancy and childhood predict adult levels of DNAm in genes that shape inflammatory phenotypes implicated in cardiovascular diseases. A later study [209], using methylation data of the same cohorts, found that low socio-economic status (SES), from infancy to young adulthood, predicts the DNAm levels of 2546 CpG sites. These CpG sites were found across 1537 genes that are involve biological pathways related to immune function and development of the nervous system.

Thus, these studies suggest that early social experiences can influence DNAm of genes involved in physiological processes that contribute to the development inflammation-related diseases later in life.

### Biomarkers and future perspective

Starting from the 1980s, the necessity for valid and reliable biomarkers of ageing became evident in the pursuit of comprehending, slowing, stopping, and potentially reversing the ageing process. Recognizing the limitations of chronological age as a flawed proxy for ageing, Baker and Sprott [210] suggested pinpointing biomarkers capable of precisely and swiftly forecasting an individual’s or an organ’s functional capability and its evolution over the life span. In essence, their proposal aimed to identify markers representing biological age rather than relying solely on chronological age [210]. Among the various measures, epigenetic clocks are the most promising biomarkers of biological ageing. Epigenetic clocks are mathematical models built on the combinations of DNAm levels of CpG sites across the genome and a significant body of literature offers compelling evidence regarding the ability of these biomarkers to encompass facets of biological ageing [211]. To date, several DNAm-based epigenetic clocks have been developed through various methods. The “first-generation” clocks (*Horvath* [212] and *Hannum* [213]) were designed to estimate chronological age; the “second-generation” clocks (*PhenoAge* [214] and *GrimAge* [215]) were specifically developed to forecast health-related outcomes and predict the time to death; whereas the third-generation epigenetic clock (*DunedinPACE* [216]) was intended to track and comprehend the impact of ageing interventions along with how changes in disease conditions or lifestyle choices influence epigenetic ageing.

Furthermore, these clocks are applicable across all types of DNA sources (ranging from sorted cells to various tissues and organs) and encompass the entire age range (from prenatal tissue development to tissues found in centenarians) [212]. In general, a positive discrepancy between biological age, as estimated by these clocks, and the chronological age, indicates that the underlying tissue/organs ages faster than expected.

Although many studies investigated the effect of early life adversity on the epigenetic clocks [217–219], how the social environment and the social interactions can impact on them is still poorly understood. One remarkable finding on this topic came from the paper of Hillman and colleagues [220]. In this study, using data from Health and Retirement Study (a nationally representative study of adults over the age of 50 and their spouses), the authors found that positive social factors slow biological ageing. Specifically, their study demonstrated that increased support from friends and more frequent contact with friends were associated to a decelerated Pace of Ageing, as assessed by the *DunedinPACE* clock. Additionally, they observed that greater contact frequency with children correlated with a reduced *GrimAge*, and that an uptick in family contact over time was connected to a decreased *Hannum* age [220].

In addition to epigenetic clocks [221, 222], future research could explore other biomarkers that assess the impact of sociality on ageing. Potential biomarkers include inflammatory markers like C-reactive protein, interleukin-6 (IL-6), and other inflammatory cytokines, which are indicative of chronic inflammation—a condition linked to social isolation and stress [223, 224]. Telomere length, a marker of cellular ageing and biomarkers of oxidative stress, could also be evaluated in relation to social interactions, as chronic stress and isolation have been associated with telomere shortening and increased oxidative macromolecular damage [225]. Cortisol levels, reflecting stress-induced endocrine responses, can offer insights into the physiological toll of social stress across different life stages [226, 227]. Neuroimaging studies of brain regions involved in social cognition and stress regulation, such as the amygdala and prefrontal cortex, may also provide valuable insights into how social environments shape biological ageing processes [228].

As our knowledge of epigenetic clocks and other biomarkers of ageing advances, these tools offer a valuable unique opportunity to inform and guide public health and social policies aimed at promoting healthy ageing. Policymakers could harness longitudinal data on DNA methylation and other biological markers to design tailored interventions that address distinct ageing trajectories within different socio-economic and cultural groups and communities. In particular, in post-industrial societies, epigenetic clocks could serve as a critical measure to evaluate the effectiveness of social interventions guided by evidence-based policies. Furthermore, policies that provide support from prenatal stages through childhood and into old age could significantly reduce the risk of chronic diseases linked to accelerated biological ageing. Indeed, research has shown that ageing processes begin early in life, shaped by factors such as prenatal and early childhood environments. By addressing these early influences, such policies can promote healthier ageing trajectories and mitigate long-term health risks.

In this perspective, further studies aimed at exploring the influence of pro-social behaviours and social interactions on epigenetic clocks are essential. These studies can pave the way for evidence-based social policies that facilitate healthy ageing within a rapidly evolving social environment.

## Conclusions

In this review, we have summarized with a bioanthropological perspective the profound relationship between sociality and longevity. We show that social dynamics can shape various aspects of physiology, including neuronal, cardiovascular, endocrine and immune systems at the cellular and molecular levels, all converging on the mechanisms of stress response that, in turn, influence lifespan. We provide evidence on how sociality can influence biological mechanisms across different timescales, impacting, at different epochs of life, human ageing, and lifespan. We conclude that lifespan and healthy ageing result from the complex interplay between genetic/physiological determinants and socio-ecological factors at both individual and population levels. This complex regulatory network contributes, on an evolutionary timescale, to different life-history trajectories and lifespan variations. We emphasize the importance of interdisciplinary research capable of integrating all the above-mentioned dimensions with the final goal of identifying potential interventions to improve human healthy ageing in rapidly evolving social environment.
